# Co-Expression Network and Pathway Analyses Reveal Important Modules of miRNAs Regulating Milk Yield and Component Traits

**DOI:** 10.3390/ijms18071560

**Published:** 2017-07-18

**Authors:** Duy N. Do, Pier-Luc Dudemaine, Ran Li, Eveline M. Ibeagha-Awemu

**Affiliations:** 1 Agriculture and Agri-Food Canada, Sherbrooke Research and Development Centre, Sherbrooke, QC J1M 0C8, Canada; Do.DuyNgoc@agr.gc.ca (D.N.D.); pier-luc.dudemaine@agr.gc.ca (P.-L.D.); ran.li1986@hotmail.com (R.L.); 2 Department of Animal Science, McGill University, 21111, Lakeshore Road, Ste-Anne-de Bellevue, QC H9X 3V9, Canada; 3 College of Animal Science and Technology, Northwest A&F University, Xi’an 712100, China

**Keywords:** co-expression network, dairy cows, hub genes, lactation, microRNA, signaling pathways, transcription factors

## Abstract

Co-expression network analyses provide insights into the molecular interactions underlying complex traits and diseases. In this study, co-expression network analysis was performed to detect expression patterns (modules or clusters) of microRNAs (miRNAs) during lactation, and to identify miRNA regulatory mechanisms for milk yield and component traits (fat, protein, somatic cell count (SCC), lactose, and milk urea nitrogen (MUN)) via miRNA target gene enrichment analysis. miRNA expression (713 miRNAs), and milk yield and components (Fat%, Protein%, lactose, SCC, MUN) data of nine cows at each of six different time points (day 30 (D30), D70, D130, D170, D230 and D290) of an entire lactation curve were used. Four modules or clusters (GREEN, BLUE, RED and TURQUOISE) of miRNAs were identified as important for milk yield and component traits. The GREEN and BLUE modules were significantly correlated (|*r*| > 0.5) with milk yield and lactose, respectively. The RED and TURQUOISE modules were significantly correlated (|*r*| > 0.5) with both SCC and lactose. In the GREEN module, three abundantly expressed miRNAs (miR-148a, miR-186 and miR-200a) were most significantly correlated to milk yield, and are probably the most important miRNAs for this trait. DDR1 and DDHX1 are hub genes for miRNA regulatory networks controlling milk yield, while HHEX is an important transcription regulator for these networks. miR-18a, miR-221/222 cluster, and transcription factors HOXA7, and NOTCH 3 and 4, are important for the regulation of lactose. miR-142, miR-146a, and miR-EIA17-14144 (a novel miRNA), and transcription factors in the SMAD family and MYB, are important for the regulation of SCC. Important signaling pathways enriched for target genes of miRNAs of significant modules, included protein kinase A and PTEN signaling for milk yield, eNOS and Noth signaling for lactose, and TGF β, HIPPO, Wnt/β-catenin and cell cycle signaling for SCC. Relevant enriched gene ontology (GO)-terms related to milk and mammary gland traits included cell differentiation, G-protein coupled receptor activity, and intracellular signaling transduction. Overall, this study uncovered regulatory networks in which miRNAs interacted with each other to regulate lactation traits.

## 1. Introduction

MicroRNAs (miRNAs) are small noncoding RNA molecules about 22 nucleotides long which regulate gene expression post-transcriptionally, and play key roles in a wide range of biological processes. Many lines of evidence indicate that several miRNAs can work together to affect target genes in the same or different biological pathways [1,2,3]. Complex relationships exist between miRNAs, since they (1) might be clustered together both by sequence similarity and genomic location, (2) might be clustered into the same miRNA family, (3) may regulate or are regulated by the same transcription factor, and (4) might share target genes in certain biological processes [3,4]. Indeed, several approaches have been proposed to explore these relationships, such as miRNA–miRNA synergistic network [5] and co-expression analyses [6,7]. The first approach is based on the downstream study of miRNA target genes through construction of networks based on different weighted methods on common target genes, as well as the interactions among them [3,5]. Meanwhile, weighted co-expression network is based on construction of interaction networks (modules) of miRNAs with similar expression patterns, whereby miRNAs in the same module interact with one another to regulate the same or similar biological processes [3,8]. Moreover, every module is assigned an eigenvalue, which enables determination of the relationship (correlation) between modules and traits of interest. The hub genes of each module points to the most active miRNAs in each network, which are potentially the most important miRNAs regulating the transcriptomic mechanisms underlying the traits.

Lactation is a complex process known to be controlled by various regulatory mechanisms, including miRNAs [9,10]. The roles of miRNAs in dairy lactation or mammary gland development have been investigated by several authors [11,12,13,14,15,16,17,18]. Recently, we reported that 58 miRNAs were dynamically and differentially expressed across lactation stages, and that 19 miRNAs were differentially expressed throughout lactation in a significant and time-dependent manner [19]. These results suggest that miRNAs might interact with each other to regulate gene expression throughout lactation. However, it was not clear if these miRNAs could interact with each other to regulate lactation phenotypes, and what mechanisms underlie possible interactions. This study therefore aimed to (i) characterize groups (modules) of high interacting miRNAs during an entire bovine lactation curve using weighted gene co-expression network analysis (WGCNA), (ii) correlate important modules with milk yield and component traits, and (iii) enrich target genes of miRNAs from important modules, for exploration of the signaling pathways and upstream transcriptional regulators of milk yield and component traits.

## 2. Results

### 2.1. Milk Component Yield Trend during a Lactation Curve

Quarter and interaction between quarter and day had no significant effects on milk yield and milk components, while day significantly affected (*p* < 0.05) milk yield and milk components (except Fat%, *p* = 0.132) (Table 1). Milk production was similar from D30 up to D170, and decreased significantly by D230 and D290 (*p* < 0.001). Except for D70, Protein% on other days numerically increased slightly with significantly (*p* < 0.05) higher values by D230 (3.22 ± 0.29%) and D290 (3.57 ± 0.40%) as compared to D30 (2.98 ± 0.29%). Similarly, somatic cell count (SCC) increased numerically being significant (*p* < 0.05) on D230 and D290 as compared to D30. On the contrary, Lactose% decreased significantly (*p* < 0.01) on D230 (3.16 ± 1.21%) and D290 (3.33 ± 0.93%) as compared to D30 (4.36 ± 0.17%). There was no clear trend for milk urea nitrogen (MUN) content (mg/dL), since it had highest numerical increase on D170 (*p* < 0.05) but remained similar across all other time points throughout lactation.

### 2.2. Important miRNA Modules for Milk and Component Yields

**Figure 1:**
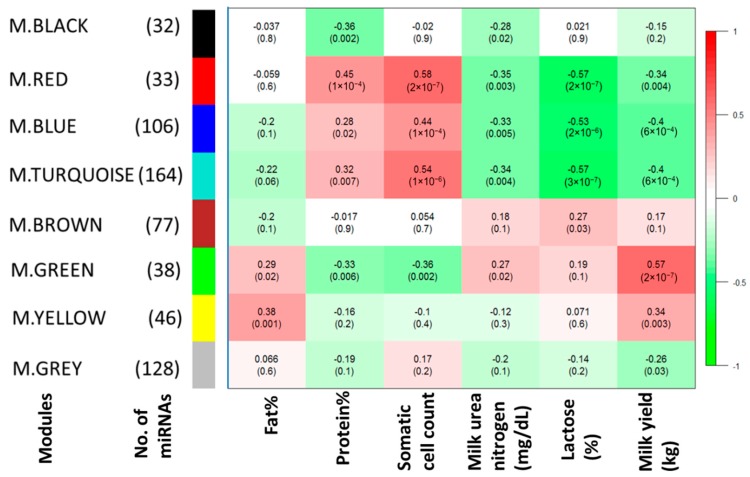
Module trait relationship: A matrix with the Module-Trait Relationships (MTRs) (correlation coefficients) and corresponding p-values (in brackets) between modules on the y-axis and lactation traits on the x-axis. The MTRs are colored based on their correlation: red is a strong positive correlation, while green is a strong negative correlation.

**Figure 2:**
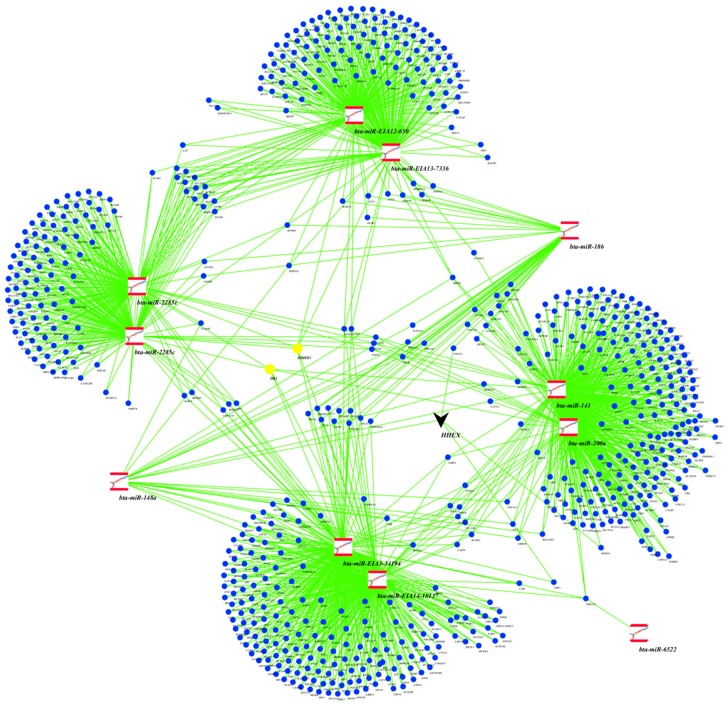
Predicted target genes of miRNAs in the GREEN module, and important hub genes and transcription factors. The red square nodes are miRNA members of the GREEN module, the blue round nodes are common predicted target genes for these miRNAs, the yellow round nodes are most highly predicted common (hub) target genes for these miRNAs, and the black V shape is the transcription regulator targeted by miRNAs, which also targets other predicted target genes in the networks.

Using the WGCNA approach, we identified eight different modules of co-expressed miRNAs, which were assigned different colors (Figure 1). miRNA membership in the modules ranged from 32 (BLACK module) to 164 (TURQUOISE module) (Figure 1). Results of the correlations between module eigengene values with traits are shown in Figure 1. Four modules (BLUE, GREEN, RED and TURQUOISE) were each significantly correlated with at least one trait (|*r*| > 0.5). A positive correlation was found between the GREEN module and milk yield (*r* = 0.57 and *p* = 2 × 10^−7^). Two modules, RED and TURQUOISE, were significantly negatively (*r* = −0.57, *p* = 2 × 10^−7^ and *r* = −0.57, *p* = 3 × 10^−7^) and positively (*r* = 0.58, *p* = 2 × 10^−7^, *r* = 0.54, *p* = 1 × 10^−6^) correlated with lactose and SCC, respectively (Figure 1). The BLUE module was significantly negatively (*r* = −0.53, *p* = 2 × 10^−6^) correlated with lactose (Figure 1). No module was significantly correlated with Fat%, Protein% and MUN. Shared target genes of members (11 miRNAs) of the GREEN module are shown in Figure 2. Numbers of miRNAs with module membership values >0.8 were 34, 11, 39 and 15 for the BLUE, GREEN, TURQUOISE and RED modules, respectively. Details of significant module memberships and correlations of modules with traits are shown in Table 2 (GREEN module), Table 3 (BLUE module), Table 4 (RED module) and Table 5 (TURQUOISE module).

### 2.3. Target Genes of miRNA Members in BLUE, GREEN, TURQUOISE and RED Modules

**Figure 3:**
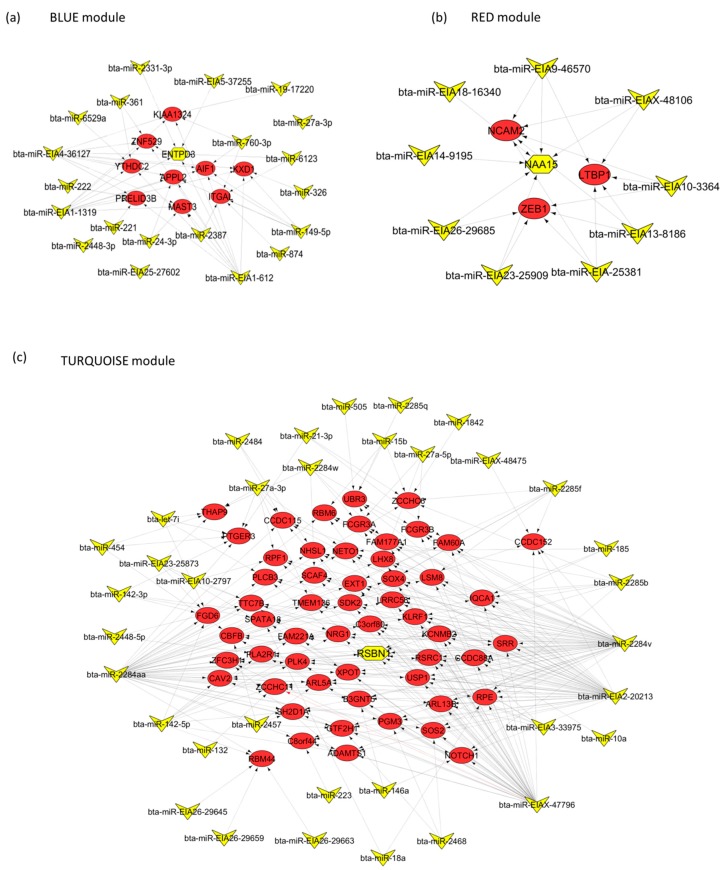
Genes targeted by at least four miRNAs in the (**a**) BLUE, (**b**) RED and (**c**) TURQUOISE modules. The yellow V-like shape represents miRNAs, red round shape represents target genes, and the hub gene (most common target) is in the center, and is represented by a hexagon shape (yellow).

The miRNA members of the BLUE, GREEN, TURQUOISE and RED modules were predicted to target 3361, 1232, 4241 and 979 unique genes, respectively (Table S1a–d). Many miRNAs shared the same target genes, especially when they had the same seed region (Table 2, Table 3, Table 4 and Table 5). The common target genes shared among members of the GREEN module are shown in Figure 2, and of the other three modules in Figure 3a (BLUE module), Figure 3b (RED module) and Figure 3c (TURQUOISE module). *DDHD1* and *DR1* genes were the most common targets for the GREEN module members, since they were each targeted by six miRNAs (Figure 2). *MIER1*, *RNPEP* and *YME1L1* genes were also targeted by five different miRNAs in the GREEN module. Meanwhile, *ENTPD3*, *RSBN1* and *NAA15* genes were the most common targets in the BLUE (targeted by six miRNAs), TURQUOISE (targeted by seven miRNAs) and RED (targeted by six miRNAs) modules, respectively (Figure 3a–c and Table S1).

### 2.4. Enriched Gene Ontologies for Target Genes of miRNA Members of the BLUE, GREEN, TURQUOISE and RED Modules

**Figure 4:**
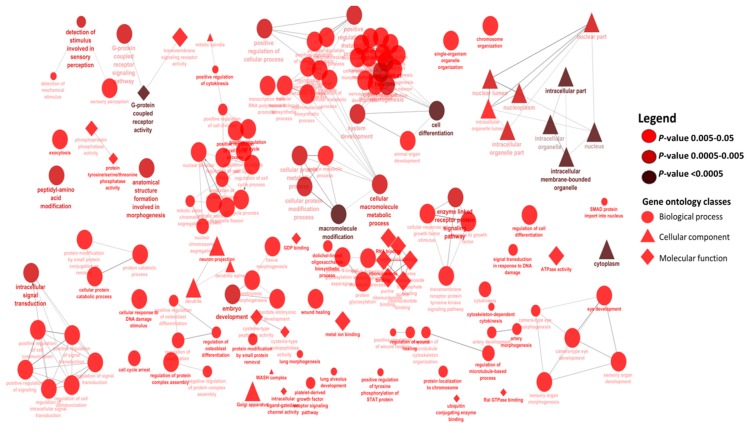
Enriched gene ontology terms for target genes of miRNAs in the GREEN module. The round, triangle and diamond shapes present biological process, cellular component and molecular function gene ontology terms, respectively.

The number of gene ontology (GO)-terms enriched for the BLUE, GREEN, TURQUOISE and RED modules were 158, 141, 174, and 65, respectively (Table S2a–d). Among them, 11 GO-terms were common to all the groups (Figure S1). The scatter plots of enriched GO-terms for the GREEN, BLUE, RED and TURQUOISE modules are shown in Figure 4 and Figures S2–S4, respectively. In the GREEN module, cell differentiation (*p* = 3.2 × 10^−4^), intracellular components (*p* = 9.6 × 10^−10^), and G-protein coupled receptor activity (*p* = 3.7 × 10^−5^) were the most significantly enriched biological process, cellular component, and molecular function GO-terms, respectively (Figure 4, Table S2b). The BLUE and TURQUOISE modules shared three most significantly enriched GO-terms, which were sensory perception of chemical stimulus, intracellular part and G-protein coupled receptor activity for biological process, cellular component and molecular function, respectively (Figures S2 and S4, and Table S2a,c). For the RED module, cellular component morphogenesis (*p* = 3 × 10^−3^), cytoplasm (*p* = 1.1 × 10^−5^) and *N*-acyltransferase activity (*p* = 3.7 × 10^−2^) were the most significantly enriched biological process, and cellular component and molecular function GO-terms, respectively (Figure S3 and Table S2d).

### 2.5. Signaling Pathways and Transcription Factors Enriched for miRNA Members of the BLUE, GREEN, TURQUOISE and RED Modules

**Figure 5:**
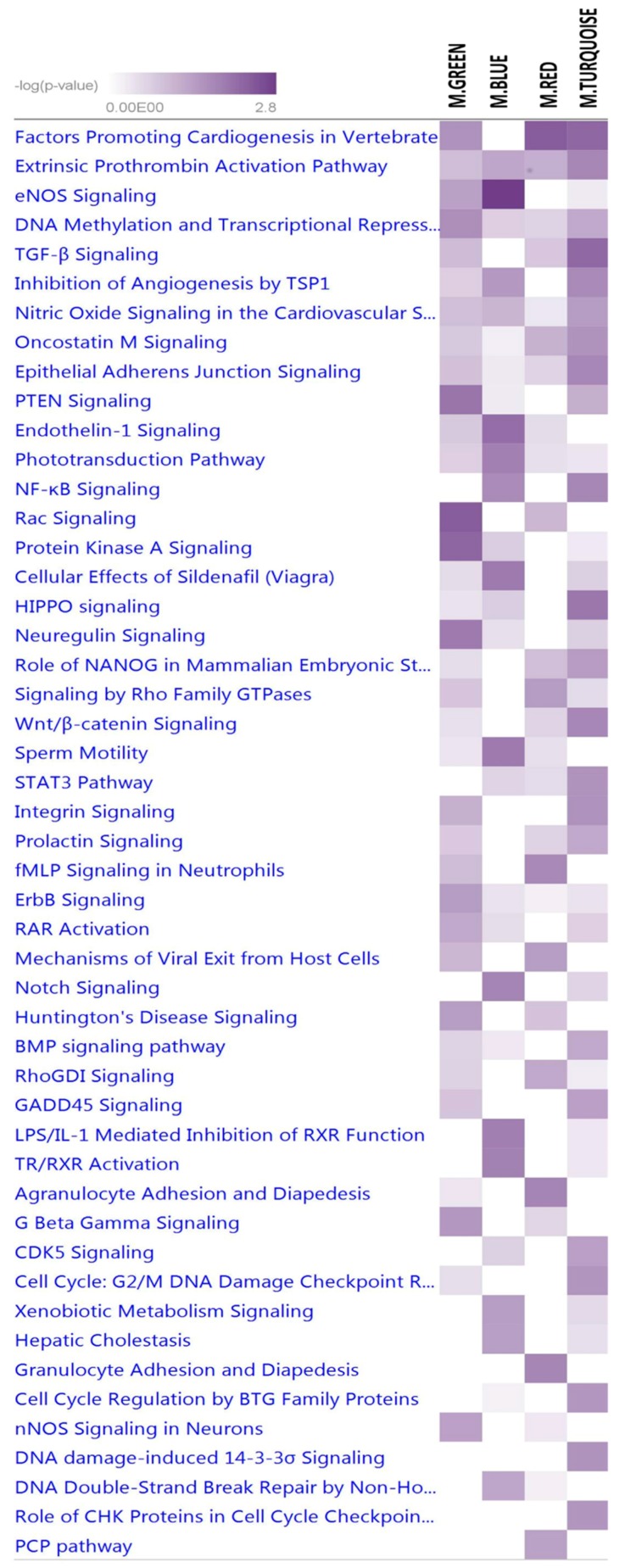
Enriched signaling pathways for target genes of miRNAs in the GREEN, BLUE, RED and TURQUOISE modules.

A total of 18, 15, 21 and 11 canonical signaling pathways were significantly enriched for the target genes predicted for 34, 11, 39 and 15 miRNA members of the BLUE, GREEN, TURQUOISE and RED modules, respectively, using IPA core analysis (Figure 5 and Table S3a–d). Rac, PTEN and protein kinase A signaling pathways were the most significantly enriched for target genes of miRNA members of the GREEN module (Table S3a), and consequently are the most significant pathways for milk yield. Other enriched relevant pathways in the module for milk yield included 3-phosphoinositide biosynthesis, RAR activation, and signaling pathways of PTEN, ErbB, DNA methylation and transcriptional repression, eNOS, and Neuregulin (Figure 5, Table S3a). Meanwhile, eNOS and Endothelin-1 signaling were the most significantly enriched for the BLUE module (Table S3b). Other relevant pathways for the BLUE module included NF-κB signaling, Notch signaling, phototransduction, and TR/RXR activation pathways. In the TURQUOISE module, several pathways related to cell cycle checkpoint were enriched (Table S3c); meanwhile, several pathways involved in nucleic acid metabolism were significantly enriched for the RED module (Table S3d).

A total of 35, 13, 99 and 9 transcription factors were enriched for target genes of miRNA members of the BLUE, GREEN, TURQUOISE and RED modules, respectively (Table 6 and Table S4). The transcription factor *HHEX* was targeted by miR-148a. Moreover, *HHEX* also regulates genes (*VEGFA*, *NRP1* and *MYH10*) which are targeted by other miRNAs in the GREEN module (Figure 2). The significantly enriched transcription factor, *SMADI*, was targeted by several miRNAs in the RED module. *SMADI* also regulates several miRNA target genes (*COL4A1*, *CXCL2*, *HHEX* and *PDGFB*) in the RED module. Seven (*ATXN1*, *NOTCH3*, *CTNNB1*, *NOTCH4*, *EOMES*, *KLF2* and *BHLHE22*) and 12 (*SP3*, *YAP1*, *BACH1*, *SMAD7*, *EHF*, *STAT3*, *KLF4*, *CDKN2B*, *BMI1*, *SMAD4*, *E2F8* and *E2F7*) transcription factors were directly targeted by miRNA members of the BLUE and TURQUOISE modules, respectively (Table 6 and Table S4).

## 3. Discussion

### 3.1. Milk Yield and Components during Lactation

The characteristics of milk yield during a bovine lactation curve are well documented. Milk yield increases from the first day of lactation, to peak milk (around day 60), followed by a gradual decrease until the end of lactation [20,21]. Previously, we have shown that milk yield of cows in this study followed a similar pattern expected of a standard lactation curve [19], such as in Wood’s model [22]. Significant variations by day recorded for milk components (except for Fat%) is supported by previous studies, which reported significant variations for milk component traits by lactation day, and also by periods of lactation [23,24,25,26]. Nevertheless, the number of phenotypic records in our data was small, which might explain the non-significant effect of day observed for milk Fat% in this study.

### 3.2. miRNAs, Hub Target Genes, Gene Ontologies, Pathways and Transcription Factors Involved in Milk Yield

A notable result in this study was the significant positive correlation (*r* = 0.57, *p* = 2 ×10^−7^) between the GREEN module and milk yield. Some miRNA members of the GREEN module are known to have potential roles in various aspects related to milk yield, including mammary gland development, such as miR-200a [27,28], immune response and epigenetic upregulation, such as miR-148a [29,30,31], or nutrient response, such as miR-141 [18]. In fact, three miRNAs (miR-148a, miR-186 and miR-200a) that displayed the highest positive correlations with milk yield (*r* = 0.52–0.54) (Table 2) are among the most abundantly expressed miRNAs in mammary gland tissues or milk [18,19,32,33], suggesting important roles for these miRNAs during lactation [34]. For instance, both miR-148a and miR-200a are known to be involved in the regulation of cell proliferation and death [28,35,36,37], crucial processes for the regulation of milk yield. Recently, Melnik et al. [30] reviewed the epigenetic regulatory roles of miR-148a in lactation, and suggested that bovine miR-148a could be a critical factor for human health, since it is a component of milk fat globule and milk exosomes and highly expressed in milk, as well as having an identical seed region with human miR-148a. Moreover, 8 of the 11 miRNAs with module membership values >0.8 in the GREEN module were predicted to target two hub genes (*DR1* and *DDHD1)* (Figure 2). Interestingly, *DR1* (encodes a TATA box-binding protein associated phosphoprotein that represses RNA polymerase II [38]) was also predicted to be targeted by miR-2285e, miR-2285c, miR-141, and miR-200a. Although no direct association has been reported between *DR1* and milk yield up to now, its role in gene transcription regulation suggest that *DR1* is an important hub gene for miRNA network regulation of milk yield. *DDHD1* gene, a member of the intracellular phospholipase family, encodes phospholipase enzyme, and is involved in catalyzing the degradation of phosphatidic acid, as well as attenuating cell activation [39]. The *DDHD1* gene might play roles in milk yield via its roles in various biological processes, such as phospholipid metabolism and related signaling pathways [40]. Further supporting evidence for the role of GREEN module miRNAs in milk yield came from the enrichment results of their target genes. Gene ontology enrichment showed the importance of cell differentiation (*p* = 3.2 × 10^−4^) in milk yield, since it was the most significantly enriched biological process GO-term (Figure 4). Moreover, intracellular signaling transduction and embryo development were also among the most significant enriched biological processes. This supports the relationship between reproduction and milk yield in cows [41,42,43], since most lactating dairy cows are also gestating. Similarly, signaling pathways important for mammary gland development, as well as for lactation, such as GO-term intracellular signaling transduction, were highly enriched for milk yield [44]. Several known and notable signaling pathways for milk yield were enriched including PTEN, prolactin, Rac, protein kinase A, neuregulin, ErbB, and TGF-β signaling (Figure 5). The roles of these pathways have been discussed in our previous study on the same animals [19]. For instance, prolactin and PTEN signaling are important pathways for hormone regulation of milk secretion [44,45], while TGF-β might have roles in mammary gland health [46,47] which is important for the maintenance of milk yield.

It is well documented that miRNAs and transcription factors can co-regulate gene expression [48,49,50]. However, little is known about the interaction of transcription factors and miRNAs in lactation regulation in general, and in milk yield regulation in particular. Enrichment of transcription factors for target genes of miRNAs in the GREEN module identified significantly enriched transcription factors, such as *EHMT2*, *ZNF350*, *HHEX*, *MITF*, *CCND1*, *TP53*, *SP1*, *RYBP*, *TAL1*, and members of SMAD family, including *SMAD* 2, 3, and 7. Interestingly, miR-148a regulates *HHEX*, while *HHEX* regulates several genes, including *VEGEA* (targeted by miR-186), *NRP1* (targeted by miR-148a), or *MYH10* (targeted by miR-141 and miR-200a) in the GREEN module (Figure 2). *HHEX* plays an important role in the development of multiple organs, including liver, thyroid, and forebrain, via interaction with other signaling molecules [51]. Using cell culture experiments, Puppin et al. [52] observed that modification of HHEX protein localization occurs during lactation and tumorigenesis, and further suggested that *HHEX* may play a role in differentiation of mammary epithelial cells. However, there has been no functional study on the role of *HHEX* in lactation and milk production. Other transcription factors like *CCND1*, *RYBP*, and *TP53* have been reported to play important roles in cellular cycle regulation and related processes [53,54,55], so they can either function as mediators or regulators of miRNA networks controlling milk yield. Furthermore, the regulation of milk fat synthesis might be an interplay between transcription factors and miRNAs (as well as other non-coding RNAs), and since these relationships are temporal and spatial, each type of molecule may have dominant roles at certain time points. Further studies, on the expression profile of transcription factors and milk at different time points throughout a lactation curve, may provide more clues about these relationships.

### 3.3. miRNAs, Hub Genes, Gene Ontologies, Pathways, and Transcription Factors Regulating Milk Components

Fat content did not change significantly during the course of lactation (Table 1), and no miRNA module was significantly correlated with Fat% and MUN. Meanwhile, lactose was significantly correlated with three different modules (RED, BLUE, and TURQUOISE). miR-18a in the BLUE module was the most significantly correlated to lactose (*r* = −0.65) (Table 3). miR-18a is a member of miR-17/92 cluster, and plays a role in tumor progression [56,57]. For example, miR-18a impairs cancer cell growth via inhibition of the expression of *CDC42* gene [56], or negatively regulates the expression of *PIAS3*, an inhibitor of *STAT3* [58]. Wu et al. [58] reported that the expression of miR-18a was positively correlated with the expression of *STAT3* gene in gastric adenocarcinoma tissues. These authors further indicated that the correlated expression was via a down-regulation of *PIAS3* gene by miR-18a. Moreover, Cai et al. [59] reported that promoter binding of STAT3 is required for transactivation of miR-148a (and other members of miR-17/92 cluster) in human macrophage cells following Toxoplasma infection. According to Brock et al. [60], a promoter region of miR-18a and other members of miR-17/92 contain a functional binding site for STAT3, which transcriptionally activates these miRNAs. However, it is not clear how miR-18a regulates milk lactose metabolism, but we speculate that its regulatory role is via targeting important molecules involved in lactation, such as STAT3.

Some notable members of the BLUE module, such as members of miR-221/222 cluster, are involved in breast cancer cell regulation by targeting multiple pathways [61], such as those promoting epithelial-to-mesenchymal transition [62]. miR-221 also targets *PTEN* [63], an important transcription factor in lactation regulation [44]. *PTEN* is known to regulate mammary cell growth, proliferation, and survival, by down-regulating important pathways of lactation, such as PI3K-AKT and mitogen-activated protein kinase pathways [64]. For instance, *PTEN* can down-regulate PTEN-AKT pathway, which is required for the initiation of lactation through the induction of autocrine prolactin [65]. Overexpression of *PTEN* was shown to down-regulate the expression of *MAPK*, *CCND1*, *AKT*, *MTOR*, *S6K1*, *STAT5*, *SREBP1*, *PPARγ*, *PRLR*, and *GLUT1* genes, or up-regulate *EIF4EBP1* gene, which are important for lactation related signaling pathways [44]. Moreover, Wang et al. [32] observed an increase in the expression of miR-221 during lactation, and suggested a role in the control of endothelial cell proliferation or angiogenesis. The most significantly enriched biological process GO-term for target genes of BLUE module miRNAs, was “sensory perception of chemical stimulus”, required for an organism to receive a sensory chemical stimulus, convert it to a molecular signal, and recognize and characterize the signal [66]. Interestingly, the most significant molecular function GO-term was “G-protein coupled receptor activity”, which involves a combination of extracellular signaling and signal transduction across membranes by activation of associated G-protein [66]. Wickramasinghe et al. [67] reported that “G-protein coupled receptor activity” was significantly enriched for differentially expressed genes at the peak of lactation. G-protein coupled receptors play roles in mediating oxytocin hormone [68], an important hormone for mammary gland cell differentiation and lactation [69,70,71]. Interestingly, both signaling pathways and upstream transcription regulator enrichments identified Notch gene family (Table 6) and Notch signaling pathway (Figure 4) as important for milk lactose metabolism. Notch signaling is also important for mammary gland development and lactation [72], as it controls mammary epithelial cell fate [73]. Moreover, Notch 3 and 4 were also targeted by miR-874 and miR-2331-3p in the BLUE module, respectively. *HOXA7*, the most enriched upstream transcription regulator in the BLUE module, regulates the transcription of several genes including *CD93*, *EDNRA*, *EGFR*, *IL7R*, *KCNA3*, *MSI2*, *PGR*, *TSC22D1*, and *SOX4* (a master regulator of epithelial–mesenchymal transition [74]). These results support the roles of BLUE module miRNAs in epithelial–mesenchymal transition.

The RED module showed significant correlation with two traits, SCC and lactose. However, since all members of the RED module, except miR-2285v, are novel miRNAs, no documented functions are available for these miRNAs. The most important enriched biological processes GO-term for target genes of miRNAs in the RED module was “cellular component morphogenesis”. This term is defined as the process in which cellular structure is generated and organized, and so supports an important role of the RED module in cell regulation. Furthermore, biological processes GO-terms enriched for the RED module are involved in cell fate, such as “cell differentiation”, “cell mobility”, “cell development”, and “cell migration”. Further supporting evidence of the relationship between the RED module miRNAs and SCC, was through the significant enrichment (using IPA core analysis) of the planar cell polarity (PCP) pathway (Figure 5). The PCP pathway involves a set of core molecular regulators coordinating the orientation of cells within a tissue sheet [75,76]. Interestingly, this pathway also contributes to glucose homeostasis, which is important for lactose metabolism [77]. Activation of PCP signaling locally activates Rac signaling to regulate cell fate specification events and cellular movements [78]. Indeed, Rac signaling pathway was also significantly enriched for the RED module. Notably, *MYB* was the most significant transcription factor enriched for target genes (including *BCL2*, *CD4*, *COL4A1*, *FUT8*, *GATA3*, *ITPR1*, *KIT*, *KITLG*, *MMP1*, *MMP3*, and *SOCS2*) of miRNAs in the RED module. The *MYB* gene is well documented to play important roles in cell growth, differentiation, and apoptosis [79].

The TURQUOISE module, just like the RED module, was significantly correlated with SCC (*r* = 0.54) and lactose (*r* = 0.57). The TURQUOISE module has two hub miRNAs in the duplex form of miR-1423p and miR-1425p. It is well documented that miRNAs can form miRNA–miRNA duplexes through reverse complementary binding events if they have completely or partially complementary structures [3,80,81]. Functional analysis of miR-142-3p indicated its importance in regulating signal to balance cell cycle processes such as balance of cell proliferation and differentiation of mesenchymal cells [82]. Interestingly, miR-223 and miR-142 showed strong interaction in the TURQUOISE module. miR-223 has been shown to up-regulate miR-142 expression through transcription factors (LMO2-L/-S isoforms and CEBP-β) [83]. Moreover, miR-223 was down-regulated after lactation peak, and might play a role in the mammary response to pathogens after parturition [32]. Similar to the BLUE module, “sensory perception of chemical stimulus” and “G-protein coupled receptor activity” GO-terms were the most significant enriched biological processes and molecular function terms for the TURQUOISE module. The role of miRNAs in the TURQUOISE module in the regulation of SCC is reflected by several cell cycle pathways enriched for its miRNAs target genes, such as cell cycle G2/M DNA damage checkpoint regulation, cell cycle regulation by BTG family proteins, and the role of CHK proteins in cell cycle checkpoint control pathways (Figure 5). Moreover, many other relevant signaling pathways for mammary gland and lactation were significantly enriched, such as TGF-β, HIPPO, Wnt/β-catenin, epithelial adherent junction, NF-κB, integrin, CDK5, BMP and prolactin, as well as the STAT3 pathway. These pathways were also significantly enriched by target genes of differentially expressed miRNAs in our previous study [19]. Notably, *STAT3* and *STAT5A* (key transcription factors during lactation and mammary gland involution [84,85,86,87,88,89]) were also enriched for the target genes of miRNAs in the TURQUOISE module. Interestingly, *STAT3* is also directly targeted by miR-27a-5p of the TURQUOISE module.

## 4. Materials and Methods

### 4.1. Animal Management and Milk Sampling

Animal use and experimental procedures were according to the national codes of practice for the care and handling of dairy cattle (www.nfacc.ca) and approved by the Animal Care and Ethics Committee of Agriculture and Agri-Food Canada.

Procedures for animal management and data collection have been reported previously [19]. Briefly, nine Canadian Holstein cows, first to third parity, were used. Cows were fed a mixed ration of corn and grass silages (50:50) and concentrate, and managed following standard procedures. Animals were housed in individual tie stalls and allowed *ad libitum* access to feed and water at all times. Milk samples, about 50 mL composite of left and right back quarters of morning and evening milking, were collected on different days (D) in milk (D30, D70, D130, D170, D230, and D290) to represent an entire lactation curve for the measurement of milk components (fat (Fat%) and protein percentages (Protein%), lactose, milk urea nitrogen (MUN) and somatic cell count (SCC). Daily milk yield for each cow was recorded with electronic milk meters (MU-480, De Laval Inc. Kansas City, USA). Milk samples for RNA isolation (50 mL) were collected two hours after the morning milking at different times throughout the lactation curve; day 1 in milk [D1], D7, D30, D70, D130, D170, D230 and D290. Samples were placed on ice, transferred to the laboratory, and immediately processed to reduce potential RNA degradation. In the laboratory, milk was mixed well and centrifuged at 1900 *g* for 15 min at 4 °C. The fat layer (upper phase) was transferred to a 50 mL RNase free falcon tube and ~7.5 mL Qiazol lysis reagent (Qiagen Inc., Hilden, Germany) was added, and vigorously mixed by vortexing until the fat was well dispersed. The homogenized fat was stored at −80 °C until used.

### 4.2. Milk Component Analysis

Test-day milk Fat%, Protein%, MUN and lactose contents were measured with MilkoScan FT 6000 Series mid-range infrared Fourier transform infrared-based spectrometers (Foss, Hillerod, Denmark), while milk SCC was measured with Fossomatic flow cytometric cell counter (Fossomatic 5000, Foss electric, Hillerod, Denmark). Milk component analysis was performed by Valacta (Valacta Laboratories Inc., Ste-Anne-de-Bellevue, QC, Canada, www.valacta.com), a commercial laboratory specialized in such analyses.

### 4.3. Statistical Analysis

Statistical analysis was performed with SAS version 9.3 software (SAS Institute Inc., Cary, NC, USA). The effect of day on milk yield and components was analyzed using a two-way analysis of variance in a completely randomized design with repeated measures. The effect of day was tested with multiple comparisons to D30 using a Dunnett correction. Quarter (cow) was used as the subject of the repeated analysis, and the best variance–covariance matrix was selected using the AICC (Corrected Akaike Information Criteria) criteria for each variable analysed. ARH (I) variance–covariance matrix was used for all variables, except that CSH was used for lactose.

The statistical model included the fixed effects of quarter and day.

*Y_ijk_* = *μ* + *q_i_* + *cow_(q)_k_(i)_* + *d_j_* + *(qd)_ij_* + *eijk*, (1)

where *Y_ijk_* = observation for quarter *i* sampled on day *j* for animal *k*, *μ* = general mean, *q_i_* = fixed effect of quarter *i* = left, right, *cow_(q)_k_(i)_* = random effect (subject of the repeated measure), *d_j_* = fixed effect of time of sampling (day) *j*, *qd_ij_* = interaction between quarter and day of sampling, and *e_ijk_* = residual error. Probabilities lower than 0.05 (*p* < 0.05) were declared significant.

### 4.4. Total RNA Isolation, miRNA Sequencing, and Bioinformatics Management of Data

Procedures for RNA isolation, miRNA sequencing, and bioinformatics management of data have been reported previously [19]. In brief, total RNA was extracted from fat homogenate using miRVana miRNA isolation kit (Life Technologies, Carlsbad, CA, USA). RNA was digested with Turbo DNase (Ambion Inc., Austin, TX, USA) and purified using Zymo RNA clean and concentrator-25 kit (Zymo Research, Irvine, CA, USA). The integrity of RNA (RIN) was determined on an Agilent 2100 Bioanalyzer using RNA 6000 Pico kit (both from Agilent Technologies, Richardson, TX, USA). Isolated total RNA with RIN values from 2.3 to 8.5 and having an intact small RNA fraction on an Agilent 2100 Bioanalyzer electropherogram were used for library preparation [19]. A total of 71 libraries were prepared and subjected to 50 bp single end sequencing on an Illumina HiSeq 2000 system (Illumina Inc., San Diego, CA, USA) using TruSeq v3 reagents. The identification of known miRNAs and discovery of novel miRNAs were performed using miRDeep2 v2.0.0.8 [90], which uses a probabilistic algorithm based on the miRNA biogenesis model and designed to detect miRNAs from deep sequence reads. The expression of miRNAs (count table) was normalized using Deseq2 package (v1.11.19) [91]. Deseq2 models read count data with a negative binomial distribution. The normalized data was extracted using count () function and a final normalized matrix containing 713 miRNAs (475 known and 238 novel) [19] was used for network construction.

### 4.5. Gene Co-Expression Network Analysis

The weighted gene correlation network analysis (WGCNA) R-package [92] was used for miRNA co-expression network analysis. The input for co-expression analysis was normalized data of 713 miRNAs obtained from miRNA expression data as described above and in details in Do et al. [19]. To compute the co-expression network for whole lactation data, an adjacency matrix was generated by calculating Pearson’s correlation between all miRNAs, and raising it to a power β (soft threshold) of 7. The power value was chosen using a scale-free topology criterion (*r*^2^ = 0.95). Then, miRNAs were clustered using degree of overlap in shared neighbors between them, a topological overlap measure (TOM) was calculated and a value between 0 and 1 was assigned to each miRNA pair. When miRNAs share the same neighbors, a TOM value of 1 is assigned, and when they do not share any neighbor, a TOM value of 0 is assigned. An average linkage hierarchical clustering using the dynamic tree-cutting algorithm to produce a clustering tree (dendrogram) [93] was performed. Each branch of the tree is a module, and modules with at least 20 miRNAs were assigned a color. Details about methodology and the relative merits of WGCNA have been provided previously [92,93,94].

The module–trait relationship was used to select potential biologically interesting modules for downstream analysis. Module–trait relationship was computed based on Pearson’s correlation between the module eigengene values and milk yield and components. The eigengene value is defined as the first principal component of a given module, and it represents a measure of gene expression profiles in the module. A module was chosen for further analysis if it had a value of module–trait relationship >|0.5| for any phenotype. Furthermore, miRNAs in selected modules were used for functional enrichment analysis only if their intra-modular connectivity with the module was >0.8. Intra-modular connectivity measures how co-expressed a given gene or miRNA is with the other miRNAs (members) within the module, and can also be called module membership.

### 4.6. Function Enrichment of Target Genes of miRNAs in Significant Modules

TargetScan was used to predict the target genes of miRNAs (known and novel) in modules significantly associated with milk traits. Perl scripts from the TargetScan website (www.targetscan.org) were used to predict (targetscan_60.pl), and to calculate, the context scores (targetscan_61_context_scores.pl) of target genes. Predicted target genes with context + scores above 95th percentile were further used [18]. The target genes of module specific miRNAs were enriched for gene ontology (GO) terms using ClueGO [95], and pathways and upstream transcription regulators using Ingenuity Pathway Analysis (IPA) software (Ingenuity Systems Inc., Redwood City, CA, USA). For ClueGO analysis, a hypergeometric test was used for GO-term enrichment and Benjamini–Hochberg [23] correction for multiple testing-controlled *p*-values. For IPA core analysis, pathways were considered significantly over-represented at a Benjamini–Hochberg corrected *p*-value ≤ 0.05, and contained at least two target genes from data input.

## 5. Conclusions

Overall, this study identified regulatory networks and related mechanisms by which miRNAs interact with each other to regulate lactation phenotypes. Important hub miRNAs, transcription factors and regulatory networks for milk traits were uncovered. Moreover, the enrichment of important signaling pathways for milk yield and milk components enhances our knowledge about the regulation of milk composition at the molecular level. In addition, miRNAs demonstrated various ways of interaction, including shared common target genes, enriched transcription factors and regulatory pathways as well as formation of miRNA–miRNA duplexes in the regulation of lactation phenotypes. Further functional characterization of important module miRNAs and hub genes will further understanding of their roles, as well as inform of their potential use as biomarkers in selection programs for high milk yield or milk with specific requirements.

## Figures and Tables

**Table 1:** Milk and component yields (mean ± standard deviation) throughout the lactation curve. For each trait, data for each day was compared with day 30.

Lactation Day	Milk Yield (kg)	Fat%	Protein%	Milk Urea Nitrogent (mg/dL)	Lactose%	Log of Somatic Cell Count
Day 30	41.18 ^a^ ± 1.99	5.50 ^b^ ± 2.26	2.98 ^a^ ± 0.29	11.91 ^a^ ± 1.55	4.36 ^a^ ± 0.17	4.92 ^a^ ± 0.46
Day 70	42.50 ^a^ ± 1.86	5.63 ^b^ ± 1.61	2.77 ^a^ ± 0.28	12.46 ^a^ ± 3.60	4.05 ^a^ ± 0.70	5.23 ^a^ ± 0.82
Day 130	42.04 ^a^ ± 2.16	3.96 ^a^ ± 1.89	2.99 ^a^ ± 0.29	12.62 ^a^ ± 1.57	4.14 ^a^ ± 0.54	5.26 ^a^ ± 0.82
Day 170	38.38 ^a^ ± 1.86	5.05 ^b^ ± 1.36	3.11 ^a^ ± 0.14	16.22 ^b^ ± 5.41	4.30 ^a^ ± 0.16	5.22 ^a^ ± 1.02
Day 230	31.99 ^b^ ± 2.16	4.63 ^b^ ± 0.72	3.45 ^b^ ± 0.54	13.03 ^a^ ± 2.12	3.16 ^b^ ± 1.21	5.89 ^b^ ± 0.78
Day 290	25.69 ^b^ ± 1.86	4.46 ^a^ ± 1.08	3.57 ^b^ ± 0.40	12.85 ^a^ ± 4.85	3.33 ^b^ ± 0.93	5.88 ^b^ ± 0.91
Over all *p*-value, day effect	<0.001	0.132	<0.001	0.023	0.001	0.001

^a,b^ For each trait, means in the same column with a different superscript, differ significantly from Day 30.

**Table 2:** microRNAs (miRNAs) in the GREEN module, their correlation coefficients with milk yield, and predicted target genes.

miRNA	Module Membership (Eigenvalue)	*p*-Value of Module Membership	^1^ rMilk	*p*-Value rMilk	Total Target Genes	^2^ Unique Targets	^3^ Shared Targets
bta-miR-EIA12-6501	0.82	2.64 × 10^−8^	0.32	7.60 × 10^−3^	159	83	76
bta-miR-EIA13-7336	0.84	4.14 × 10^−20^	0.41	4.22 × 10^−4^	151	77	74
bta-miR-141	0.80	8.53 × 10^−17^	0.47	3.74 × 10^−5^	209	101	108
bta-miR-EIA14-10137	0.82	3.06 × 10^−18^	0.35	3.12 × 10^−3^	241	117	124
bta-miR-148a	0.84	2.34 × 10^−19^	0.54	1.36 × 10^−6^	162	147	15
bta-miR-186	0.82	5.08 × 10^−18^	0.58	1.67 × 10^−7^	383	362	21
bta-miR-200a	0.81	1.43 × 10^−17^	0.52	4.53 × 10^−6^	240	123	117
bta-miR-2285c	0.84	6.30 × 10^−20^	0.35	3.23 × 10^−3^	124	54	70
bta-miR-2285e	0.82	1.76 × 10^−18^	0.31	1.01 × 10^−2^	124	55	69
bta-miR-EIA3-34194	0.82	6.02 × 10^−18^	0.32	6.87 × 10^−3^	209	96	113
bta-miR-6522	0.80	5.36 × 10^−17^	0.47	4.11 × 10^−5^	18	17	1

^1^ rMilk is the Person correlation coefficient between miRNA and milk yield; ^2^ Gene targets unique to miRNA; ^3^ Gene targets shared with other miRNAs in the GREEN module.

**Table 3:** miRNAs in the BLUE module, their correlation coefficients with lactose and predicted target genes.

miRNA	Module Membership (Eigenvalue)	*p*-Value of Module Membership	^1^ rLactose	*p*-Value rLactose	Total Target Genes	^2^ Unique Targets	^3^ Shared Targets
bta-miR-EIA1-1319	0.80	4.73 × 10^−17^	−0.42	2.98 × 10^−4^	214	68	146
bta-miR-EIA11-5700	0.88	7.65 × 10^−24^	−0.43	1.78 × 10^−4^	214	62	152
bta-miR-1249	0.82	3.14 × 10^−18^	−0.50	1.15 × 10^−5^	90	75	15
bta-miR-149-5p	0.94	1.06 × 10^−34^	−0.54	1.64 × 10^−6^	33	24	9
bta-miR-EIA1-612	0.80	4.73 × 10^−17^	−0.42	2.98 × 10^−4^	209	167	42
bta-miR-18a	0.84	8.66 × 10^−20^	−0.65	1.20 × 10^−9^	91	75	16
bta-miR-EIA19-17220	0.84	1.44 × 10^−19^	−0.58	1.22 × 10^−7^	205	172	33
bta-miR-221	0.84	4.68 × 10^−20^	−0.57	3.33 × 10^−7^	111	57	54
bta-miR-222	0.90	4.11 × 10^−26^	−0.57	2.02 × 10^−7^	109	53	56
bta-miR-2323	0.88	1.02 × 10^−23^	−0.53	2.14 × 10^−6^	110	93	17
bta-miR-2331-3p	0.84	1.66 × 10^−19^	−0.45	7.94 × 10^−5^	182	153	29
bta-miR-2346	0.82	3.32 × 10^−18^	−0.55	7.62 × 10^−7^	130	111	19
bta-miR-2350	0.81	2.98 × 10^−17^	−0.38	9.99 × 10^−4^	119	101	18
bta-miR-2387	0.90	1.06 × 10^−25^	−0.43	1.89 × 10^−4^	205	174	31
bta-miR-2403	0.82	2.63 × 10^−18^	−0.51	5.67 × 10^−6^	32	28	4
bta-miR-EIA24-27575	0.82	3.31 × 10^−18^	−0.48	2.81 × 10^−5^	186	159	27
bta-miR-24-3p	0.84	5.77 × 10^−20^	−0.65	1.50 × 10^−9^	27	19	8
bta-miR-2448-3p	0.87	3.46 × 10^−22^	−0.41	3.65 × 10^−4^	99	83	16
bta-miR-EIA25-27602	0.87	3.53 × 10^−22^	−0.53	2.11 × 10^−6^	221	184	37
bta-miR-27a-3p	0.89	1.29 × 10^−24^	−0.63	6.10 × 10^−9^	248	202	46
bta-miR-326	0.89	5.73 × 10^−25^	−0.40	5.94 × 10^−4^	282	242	40
bta-miR-330	0.96	1.20 × 10^−39^	−0.53	2.37 × 10^−6^	242	210	32
bta-miR-3432a	0.87	9.03 × 10^−23^	−0.40	6.74 × 10^−4^	22	20	2
bta-miR-361	0.87	2.88 × 10^−22^	−0.57	3.17 × 10^−7^	115	94	21
bta-miR-378	0.81	1.86 × 10^−17^	−0.45	7.84 × 10^−5^	132	116	16
bta-miR-EIA4-36127	0.84	7.01 × 10^−20^	−0.45	8.53 × 10^−5^	214	59	155
bta-miR-505	0.80	8.51 × 10^−17^	−0.59	1.01 × 10^−7^	179	151	28
bta-miR-EIA5-37255	0.83	1.30 × 10^−18^	−0.38	1.16 × 10^−3^	91	80	11
bta-miR-EIA5-37953	0.86	1.26 × 10^−21^	−0.43	2.21 × 10^−4^	94	76	18
bta-miR-6123	0.86	2.44 × 10^−21^	−0.57	3.33 × 10^−7^	135	121	14
bta-miR-6529a	0.82	2.29 × 10^−18^	−0.26	3.27 × 10^−2^	114	91	23
bta-miR-EIA7-42699	0.85	3.48 × 10^−20^	−0.57	2.54 × 10^−7^	28	26	2
bta-miR-760-3p	0.82	2.77 × 10^−18^	−0.29	1.64 × 10^−2^	223	184	39
bta-miR-874	0.88	2.42 × 10^−23^	−0.54	1.36 × 10^−6^	154	131	23

^1^ rLactose is the Person correlation coefficient between miRNA and lactose content; ^2^ Gene targets unique to miRNA; ^3^ Gene targets shared with other miRNAs in the BLUE module.

**Table 4:** miRNAs in the RED module, their correlation coefficients with lactose and somatic cell count, and predicted target genes

miRNA	Module Membership (Eigenvalue)	*p*-Value of Module Membership	^1^ rLactose	*p*-Value rLactose	rCells ^2^	*p*-Value rCells	Total Target Genes	Unique Targets ^3^	Shared Targets ^4^
bta-miR-EIA10-2785	0.80	5.24 × 10^−17^	−0.37	1.50 × 10^−3^	0.31	8.16 × 10^−3^	152	142	10
bta-miR-EIA10-3364	0.94	3.05 × 10^−33^	−0.51	8.25 × 10^−6^	0.52	4.85 × 10^−6^	101	30	71
bta-miR-EIA13-8186	0.94	6.64 × 10^−33^	−0.51	5.17 × 10^−6^	0.52	3.37 × 10^−6^	101	27	74
bta-miR-EIA13-8622	0.91	2.29 × 10^−27^	−0.47	4.93 × 10^−5^	0.47	3.40 × 10^−5^	135	129	6
bta-miR-EIA14-9195	0.87	4.89 × 10^−23^	−0.49	1.87 × 10^−5^	0.51	7.49 × 10^−6^	55	27	28
bta-miR-EIA17-14144	0.94	1.98 × 10^−33^	−0.52	4.36 × 10^−6^	0.54	1.34 × 10^−6^	104	96	8
bta-miR-EIA18-16340	0.87	4.89 × 10^−23^	−0.49	1.87 × 10^−5^	0.51	7.49 × 10^−6^	55	26	29
bta-miR-2285v	0.81	3.28 × 10^−17^	−0.53	1.91 × 10^−6^	0.52	3.08 × 10^−6^	20	18	2
bta-miR-EIA23-25381	0.94	1.40 × 10^−32^	−0.51	6.35 × 10^−6^	0.52	5.05 × 10^−6^	101	39	62
bta-miR-EIA23-25909	0.87	7.42 × 10^−23^	−0.53	2.48 × 10^−6^	0.54	1.17 × 10^−6^	152	67	85
bta-miR-EIA24-26839	0.80	4.55 × 10^−17^	−0.41	4.24 × 10^−4^	0.48	2.13 × 10^−5^	11	10	1
bta-miR-EIA26-29685	0.87	1.15 × 10^−22^	−0.50	1.08 × 10^−5^	0.51	6.09 × 10^−6^	150	83	67
bta-miR-EIA8-44984	0.88	7.12 × 10^−24^	−0.47	3.76 × 10^−5^	0.50	1.21 × 10^−5^	167	162	5
bta-miR-EIA9-46570	0.94	3.15 × 10^−33^	−0.51	5.28 × 10^−6^	0.53	2.30 × 10^−6^	125	54	71
bta-miR-EIAX-48106	0.90	1.86 × 10^−26^	−0.49	1.81 × 10^−5^	0.50	8.50 × 10^−6^	125	69	56

^1^ rLactose is the Person correlation coefficient between miRNA and Lactose; ^2^ rCells is the Person correlation coefficient between miRNA and somatic cell counts; ^3^ Gene targets unique to miRNA; ^4^ Gene targets shared with other miRNAs in the RED module.

**Table 5:** miRNAs in the TUQUOISE module, their correlation coefficients with lactose and somatic cell count, and predicted target genes.

miRNA	Module Membership (Eigenvalue)	*p*-Value of Module Membership	^1^ rLactose	*p*-Value rLactose	^2^ rCells	*p*-Value rCells	Total Target Genes	^3^ Unique Targets	^4^ Shared Target Genes
bta-let-7i	0.88	3.15 × 10^−24^	−0.43	1.69 × 10^−4^	0.41	3.72 × 10^−4^	114	90	24
bta-miR-EIA10-2797	0.83	9.62 × 10^−19^	−0.39	9.88 × 10^−4^	0.39	9.26 × 10^−4^	108	81	27
bta-miR-10a	0.91	2.52 × 10^−27^	−0.49	1.67 × 10^−5^	0.54	1.12 × 10^−6^	87	67	20
bta-miR-1249	0.81	4.51 × 10^−17^	−0.50	1.15 × 10^−5^	0.54	1.69 × 10^−6^	33	27	6
bta-miR-132	0.83	8.64 × 10^−19^	−0.52	3.96 × 10^−6^	0.52	3.12 × 10^−6^	162	127	35
bta-miR-142-3p	0.93	3.20 × 10^−32^	−0.57	2.32 × 10^−7^	0.58	1.54 × 10^−6^	210	162	48
bta-miR-142-5p	0.96	1.04 × 10^−37^	−0.57	2.07 × 10^−7^	0.57	3.14 × 10^−7^	235	174	61
bta-miR-146a	0.93	2.11 × 10^−30^	−0.58	1.48 × 10^−7^	0.59	8.19 × 10^−8^	144	121	23
bta-miR-147	0.88	3.94 × 10^−24^	−0.51	7.14 × 10^−6^	0.53	2.89 × 10^−6^	23	19	4
bta-miR-15b	0.88	1.85 × 10^−23^	−0.65	1.17 × 10^−9^	0.53	2.23 × 10^−6^	215	165	50
bta-miR-1842	0.83	1.60 × 10^−18^	−0.64	2.65 × 10^−9^	0.60	5.55 × 10^−8^	227	200	27
bta-miR-185	0.80	9.73 × 10^−17^	−0.37	1.72 × 10^−3^	0.44	1.38 × 10^−4^	260	219	41
bta-miR-18a	0.83	4.36 × 10^−19^	−0.65	1.20 × 10^−9^	0.58	1.57 × 10^−7^	91	67	24
bta-miR-21-3p	0.84	2.23 × 10^−19^	−0.38	1.15 × 10^−3^	0.42	3.09 × 10^−4^	331	95	236
bta-miR-EIA2-20213	0.85	1.70 × 10^−20^	−0.41	4.11 × 10^−4^	0.44	1.16 × 10^−4^	230	196	34
bta-miR-223	0.84	1.86 × 10^−19^	−0.49	1.51 × 10^−5^	0.53	2.64 × 10^−6^	122	97	25
bta-miR-2284aa	0.87	5.10 × 10^−23^	−0.38	1.38 × 10^−3^	0.31	9.21 × 10^−3^	475	314	161
bta-miR-2284v	0.87	1.50 × 10^−22^	−0.38	1.37 × 10^−3^	0.38	1.07 × 10^−3^	334	84	250
bta-miR-2284w	0.93	8.26 × 10^−32^	−0.54	1.52 × 10^−6^	0.50	8.57 × 10^−6^	164	128	36
bta-miR-2285b	0.92	1.13 × 10^−28^	−0.43	2.31 × 10^−4^	0.41	3.80 × 10^−4^	261	205	56
bta-miR-2285f	0.92	2.26 × 10^−29^	−0.50	1.07 × 10^−5^	0.45	9.46 × 10^−5^	150	108	42
bta-miR-2285k	0.88	5.05 × 10^−24^	−0.49	1.69 × 10^−5^	0.46	7.50 × 10^−5^	24	21	3
bta-miR-2285q	0.88	7.27 × 10^−24^	−0.45	1.10 × 10^−4^	0.35	3.01 × 10^−3^	77	61	16
bta-miR-EIA23-25837	0.82	1.70 × 10^−18^	−0.48	3.20 × 10^−5^	0.46	6.50 × 10^−5^	91	71	20
bta-miR-EIA23-25873	0.83	8.46 × 10^−19^	−0.46	5.46 × 10^−5^	0.51	6.42 × 10^−6^	176	135	41
bta-miR-2448-5p	0.86	4.27 × 10^−21^	−0.53	2.39 × 10^−6^	0.47	3.40 × 10^−5^	17	12	5
bta-miR-2457	0.83	2.78 × 10^−19^	−0.61	1.55 × 10^−8^	0.53	2.07 × 10^−6^	127	98	29
bta-miR-2468	0.81	1.91 × 10^−17^	−0.38	1.34 × 10^−3^	0.40	6.71 × 10^−4^	149	108	41
bta-miR-2484	0.85	2.87 × 10^−20^	−0.46	6.14 × 10^−5^	0.50	1.24 × 10^−5^	86	66	20
bta-miR-EIA26-29645	0.80	7.64 × 10^−17^	−0.42	3.01 × 10^−4^	0.46	5.79 × 10^−5^	20	11	9
bta-miR-EIA26-29659	0.80	7.64 × 10^−17^	−0.42	3.01 × 10^−4^	0.46	5.79 × 10^−5^	20	8	12
bta-miR-EIA26-29663	0.80	7.64 × 10^−17^	−0.42	3.01 × 10^−4^	0.46	5.79 × 10^−5^	20	1	19
bta-miR-27a-3p	0.81	1.31 × 10^−17^	−0.63	6.10 × 10^−9^	0.56	4.82 × 10^−7^	248	203	45
bta-miR-27a-5p	0.91	1.35 × 10^−27^	−0.50	1.04 × 10^−5^	0.50	1.07 × 10^−5^	97	84	13
bta-miR-EIA3-33975	0.85	2.44 × 10^−20^	−0.55	6.90 × 10^−7^	0.50	8.53 × 10^−6^	251	199	52
bta-miR-454	0.84	5.80 × 10^−20^	−0.54	1.20 × 10^−6^	0.51	5.95 × 10^−6^	125	98	27
bta-miR-505	0.81	1.22 × 10^−17^	−0.59	1.01 × 10^−7^	0.49	1.38 × 10^−5^	94	79	15
bta-miR-EIAX-47796	0.87	6.77 × 10^−23^	−0.37	1.72 × 10^−3^	0.39	9.71 × 10^−4^	334	112	222
bta-miR-EIAX-48475	0.91	5.57 × 10^−28^	−0.58	1.31 × 10^−7^	0.52	4.14 × 10^−6^	147	128	19

^1^ rLactose is the Person correlation coefficient between miRNA and Lactose; ^2^ rCells is the Person correlation coefficient between miRNA and somatic cell counts; ^3^ Target genes unique to miRNA; ^4^ Target genes shared with other miRNAs in the TUQUOISE module.

**Table 6:** Enriched transcription factors for target genes of miRNAs in important modules for traits.

Module	Transcription Factor	*p*-Value	Module	Transcription Factor	*p*-Value
M.BLUE	HOXA7	1.61 × 10^−3^	M.GREEN	TP53	1.91 × 10^−2^
M.BLUE	TP53	1.62 × 10^−3^	M.GREEN	SMAD2	2.03 × 10^−2^
M.BLUE	CREBBP	2.14 × 10^−3^	M.GREEN	VAV2	2.09 × 10^−2^
M.BLUE	PAX7	2.20 × 10^−3^	M.GREEN	TAL1	3.01 × 10^−2^
M.BLUE	HHEX	7.20 × 10^−3^	M.GREEN	SMAD3	3.47 × 10^−2^
M.BLUE	SMARCA2	7.69 × 10^−3^	M.RED	MYB	8.41 × 10^−4^
M.BLUE	NFIL3	8.19 × 10^−3^	M.RED	LMO2	1.96 × 10^−2^
M.BLUE	EOMES	8.44 × 10^−3^	M.RED	HOXC6	2.01 × 10^−2^
M.BLUE	EGR2	9.00 × 10^−3^	M.RED	SMAD1	2.32 × 10^−2^
M.BLUE	YAP1	1.12 × 10^−2^	M.RED	JUNB	2.72 × 10^−2^
M.BLUE	MED13	1.20 × 10^−2^	M.RED	ARNT	3.11 × 10^−2^
M.BLUE	FOXP3	1.21 × 10^−2^	M.RED	ZNF384	3.51 × 10^−2^
M.BLUE	CTNNB1	1.60 × 10^−2^	M.RED	NACC1	3.15 × 10^−2^
M.BLUE	DDIT3	1.86 × 10^−2^	M.RED	PML	4.39 × 10^−2^
M.BLUE	BCL3	2.33 × 10^−2^	M.TURQUOISE	SMAD7	3.49 × 10^−6^
M.BLUE	MAML1	2.39 × 10^−2^	M.TURQUOISE	YY1	1.50 × 10^−4^
M.BLUE	KAT2B	2.40 × 10^−2^	M.TURQUOISE	E2F7	1.63 × 10^−4^
M.BLUE	KLF2	2.43 × 10^−2^	M.TURQUOISE	TP53	2.20 × 10^−4^
M.BLUE	ARNT	12.45 × 10^−2^	M.TURQUOISE	CCND1	2.42 × 10^−4^
M.BLUE	NFATC3	2.65 × 10^−2^	M.TURQUOISE	NFYB	3.98 × 10^−4^
M.BLUE	SREBF2	2.65 × 10^−2^	M.TURQUOISE	MED1	1.28 × 10^−3^
M.BLUE	NOTCH3	2.91 × 10^−2^	M.TURQUOISE	EHF	1.29 × 10^−3^
M.BLUE	NOTCH4	2.97 × 10^−2^	M.TURQUOISE	STAT3	1.55 × 10^−3^
M.BLUE	SP4	2.97 × 10^−2^	M.TURQUOISE	BMI1	2.40 × 10^−3^
M.BLUE	STAT3	3.38 × 10^−2^	M.TURQUOISE	YAP1	4.40 × 10^−3^
M.BLUE	TBX21	3.76 × 10^−2^	M.TURQUOISE	SMAD3	8.92 × 10^−3^
M.BLUE	CTBP2	4.61 × 10^−2^	M.TURQUOISE	MYB	9.24 × 10^−3^
M.BLUE	MAFF	4.61 × 10^−2^	M.TURQUOISE	STAT5A	1.19 × 10^−2^
M.BLUE	ATXN1	4.61 × 10^−2^	M.TURQUOISE	LHX2	1.33 × 10^−2^
M.BLUE	MAFK	4.61 × 10^−2^	M.TURQUOISE	SMAD4	1.43 × 10^−2^
M.BLUE	PAX6	4.63 × 10^−2^	M.TURQUOISE	FOXO4	1.47 × 10^−2^
M.BLUE	BHLHE22	4.84 × 10^−2^	M.TURQUOISE	E2F8	1.65 × 10^−2^
M.BLUE	MTF2	4.84 × 10^−2^	M.TURQUOISE	CDKN2B	1.65 × 10^−5^
M.BLUE	NR2C1	4.84 × 10^−2^	M.TURQUOISE	HHEX	1.65 × 10^−2^
M.BLUE	XBP1	4.89 × 10^−2^	M.TURQUOISE	RNF2	1.67 × 10^−2^
M.GREEN	EHMT2	6.15 × 10^−3^	M.TURQUOISE	BACH1	1.78 × 10^−2^
M.GREEN	ZNF350	6.33 × 10^−3^	M.TURQUOISE	SP3	2.66 × 10^−2^
M.GREEN	SMAD7	8.42 × 10^−3^	M.TURQUOISE	TOB1	3.35 × 10^−2^
M.GREEN	MITF	1.30 × 10^−2^	M.TURQUOISE	SMAD2	3.82 × 10^−2^
M.GREEN	HHEX	1.38 × 10^−2^	M.TURQUOISE	SIN3A	4.30 × 10^−2^
M.GREEN	SP1	1.60 × 10^−2^	M.TURQUOISE	HDAC1	4.30 × 10^−2^
M.GREEN	RYBP	1.80 × 10^−2^	M.TURQUOISE	HLX	4.33 × 10^−2^
M.GREEN	CCND1	1.90 × 10^−2^	M.TURQUOISE	KLF4	4.85 × 10^−2^

## Supplementary Materials

Supplementary materials can be found at www.mdpi.com/1422-0067/18/7/1560/s1.

## Disclaimer

Copyright to publications by the employees of the government of Canada, department of Agriculture and Agri-Food belong to Her Majesty, the Queen in Right of Canada as represented by the Minister of Agriculture and Agri-Food Canada.

## References

[1] Arner P., Kulyté A. (2015). MicroRNA regulatory networks in human adipose tissue and obesity. Nat. Rev. Endocrinol..

[2] Bandyopadhyay S., Bhattacharyya M. (2009). Analyzing miRNA co-expression networks to explore TF-miRNA regulation. BMC Bioinform..

[3] Xu J., Shao T., Ding N., Li Y., Li X. (2016). miRNA–miRNA crosstalk: From genomics to phenomics. Brief. Bioinform..

[4] Na Y.-J., Kim J.H. (2013). Understanding cooperativity of microRNAs via microRNA association networks. BMC Genom..

[5] Xu J., Li C.-X., Li Y.-S., Lv J.-Y., Ma Y., Shao T.-T., Xu L.-D., Wang Y.-Y., Du L., Zhang Y.-P. (2011). MiRNA–miRNA synergistic network: Construction via co-regulating functional modules and disease miRNA topological features. Nucleic Acids Res..

[6] Stäehler C.F., Keller A., Leidinger P., Backes C., Chandran A., Wischhusen J., Meder B., Meese E. (2012). Whole miRNome-wide differential co-expression of microRNAs. Genom. Proteom. Bioinform..

[7] Xiao Y., Xu C., Guan J., Ping Y., Fan H., Li Y., Zhao H., Li X. (2012). Discovering dysfunction of multiple microRNAs cooperation in disease by a conserved microRNA co-expression network. PLoS ONE.

[8] Yang Y., Han L., Yuan Y., Li J., Hei N., Liang H. (2014). Gene co-expression network analysis reveals common system-level properties of prognostic genes across cancer types. Nat. Commun..

[9] Li Z., Liu H., Jin X., Lo L., Liu J. (2012). Expression profiles of microRNAs from lactating and non-lactating bovine mammary glands and identification of miRNA related to lactation. BMC Genom..

[10] Le Guillou S., Sdassi N., Laubier J., Passet B., Vilotte M., Castille J., Laloë D., Polyte J., Bouet S., Jaffrézic F. (2012). Overexpression of miR-30b in the developing mouse mammary gland causes a lactation defect and delays involution. PLoS ONE.

[11] Li D., Xie X., Wang J., Bian Y., Li Q., Gao X., Wang C. (2015). MiR-486 regulates lactation and targets the PTEN gene in cow mammary glands. PLoS ONE.

[12] Ji Z., Dong F., Wang G., Hou L., Liu Z., Chao T., Wang J. (2015). miR-135a Targets and regulates prolactin receptor gene in goat mammary epithelial cells. DNA Cell. Biol..

[13] Wang J., Bian Y., Wang Z., Li D., Wang C., Li Q., Gao X. (2014). MicroRNA-152 regulates DNA methyltransferase 1 and is involved in the development and lactation of mammary glands in dairy cows. PLoS ONE.

[14] Feuermann Y., Kang K., Shamay A., Robinson G.W., Hennighausen L. (2014). miR-21 is under control of STAT5 but is dispensable for mammary development and lactation. PLoS ONE.

[15] Li H.-M., Wang C.-M., Li Q.-Z., Gao X.-J. (2012). MiR-15a decreases bovine mammary epithelial cell viability and lactation and regulates growth hormone receptor expression. Molecules.

[16] Tanaka T., Haneda S., Imakawa K., Sakai S., Nagaoka K. (2009). A microRNA, miR-101a, controls mammary gland development by regulating cyclooxygenase-2 expression. Differentiation.

[17] Bian Y., Lei Y., Wang C., Wang J., Wang L., Liu L., Liu L., Gao X., Li Q. (2015). Epigenetic regulation of miR-29s affects the lactation activity of dairy cow mammary epithelial cells. J. Cell. Physiol..

[18] Li R., Beaudoin F., Ammah A.A., Bissonnette N., Benchaar C., Zhao X., Lei C., Ibeagha-Awemu E.M. (2015). Deep sequencing shows microRNA involvement in bovine mammary gland adaptation to diets supplemented with linseed oil or safflower oil. BMC Genom..

[19] Do D.N., Li R., Dudemaine P.-L., Ibeagha-Awemu E.M. (2017). MicroRNA roles in signalling during lactation: An insight from differential expression, time course and pathway analyses of deep sequence data. Sci. Rep..

[20] Strucken E.M., Laurenson Y.C., Brockmann G.A. (2015). Go with the flow—Biology and genetics of the lactation cycle. Front. Genet..

[21] Miglior F., Sewalem A., Jamrozik J., Bohmanova J., Lefebvre D., Moore R. (2007). Genetic analysis of milk urea nitrogen and lactose and their relationships with other production traits in Canadian Holstein cattle. J. Dairy Sci..

[22] Wood P.D.P. (1967). Algebraic model of the lactation curve in cattle. Nature.

[23] Ng-Kwai-Hang K., Hayes J., Moxley J., Monardes H. (1984). Variability of test-day milk production and composition and relation of somatic cell counts with yield and compositional changes of bovine milk. J. Dairy Sci..

[24] Schutz M., Hansen L., Steuernagel G., Kuck A. (1990). Variation of milk, fat, protein, and somatic cells for dairy cattle. J. Dairy Sci..

[25] Gonzalo C., Carriedo J.A., Baro J.A., San Primitivo F. (1994). Factors influencing variation of test day milk yield, somatic cell count, fat, and protein in dairy sheep. J. Dairy Sci..

[26] Quist M., LeBlanc S., Hand K., Lazenby D., Miglior F., Kelton D. (2008). Milking-to-milking variability for milk yield, fat and protein percentage, and somatic cell count. J. Dairy Sci..

[27] Galio L., Droineau S., Yeboah P., Boudiaf H., Bouet S., Truchet S., Devinoy E. (2013). MicroRNA in the ovine mammary gland during early pregnancy: Spatial and temporal expression of miR-21, miR-205, and miR-200. Physiol. Genom..

[28] Nagaoka K., Zhang H., Watanabe G., Taya K. (2013). Epithelial cell differentiation regulated by MicroRNA-200a in mammary glands. PLoS ONE.

[29] Jin W., Ibeagha-Awemu E.M., Liang G., Beaudoin F., Zhao X. (2014). Transcriptome microRNA profiling of bovine mammary epithelial cells challenged with *Escherichia coli* or *Staphylococcus aureus* bacteria reveals pathogen directed microRNA expression profiles. BMC Genom..

[30] Melnik B.C., Schmitz G. (2017). Milk’s role as an epigenetic regulator in health and disease. Diseases.

[31] Muroya S., Hagi T., Kimura A., Aso H., Matsuzaki M., Nomura M. (2016). Lactogenic hormones alter cellular and extracellular microRNA expression in bovine mammary epithelial cell culture. J. Anim. Sci. Biotechnol..

[32] Wang M., Moisá S., Khan M., Wang J., Bu D., Loor J. (2012). MicroRNA expression patterns in the bovine mammary gland are affected by stage of lactation. J. Dairy Sci..

[33] Chen X., Gao C., Li H., Huang L., Sun Q., Dong Y., Tian C., Gao S., Dong H., Guan D. (2010). Identification and characterization of microRNAs in raw milk during different periods of lactation, commercial fluid, and powdered milk products. Cell. Res..

[34] Li R., Dudemaine P.L., Zhao X., Lei C., Ibeagha-Awemu E.M. (2016). Comparative analysis of the miRNome of bovine milk fat, whey and cells. PLoS ONE.

[35] Mateescu B., Batista L., Cardon M., Gruosso T., de Feraudy Y., Mariani O., Nicolas A., Meyniel J.-P., Cottu P., Sastre-Garau X., Mechta-Grigoriou F. (2011). MiR-141 and miR-200a act on ovarian tumorigenesis by controlling oxidative stress response. Nat. Med..

[36] Guo S.-L., Peng Z., Yang X., Fan K.-J., Ye H., Li Z.-H., Wang Y., Xu X.-L., Li J., Wang Y.-L. (2011). MiR-148a promoted cell proliferation by targeting p27 in gastric cancer cells. Int. J. Biol. Sci..

[37] Aydoğdu E., Katchy A., Tsouko E., Lin C.-Y., Haldosén L.-A., Helguero L., Williams C. (2012). MicroRNA-regulated gene networks during mammary cell differentiation are associated with breast cancer. Carcinogenesis.

[38] Inostroza A., Mermelstein F.H., Ha I., Lane W.S., Reinberg D. (1992). Dr1, a TATA-binding protein-associated phosphoprotein and inhibitor of class II gene transcription. Cell.

[39] Higgs H.N., Han M.H., Johnson G.E., Glomset J.A. (1998). Cloning of a phosphatidic acid-preferring phospholipase A1 from bovine testis. J. Biol. Chem..

[40] Richmond G.S., Smith T.K. (2011). Phospholipases A1. Int. J. Mol. Sci..

[41] Patton J., Kenny D., McNamara S., Mee J., O’mara F., Diskin M., Murphy J. (2007). Relationships among milk production, energy balance, plasma analytes, and reproduction in Holstein-Friesian cows. J. Dairy Sci..

[42] Hansen L. (2000). Consequences of selection for milk yield from a geneticist’s viewpoint. J. Dairy Sci..

[43] Buckley F., O’sullivan K., Mee J., Evans R., Dillon P. (2003). Relationships among milk yield, body condition, cow weight, and reproduction in spring-calved Holstein-Friesians. J. Dairy Sci..

[44] Wang Z., Hou X., Qu B., Wang J., Gao X., Li Q. (2014). *Pten* regulates development and lactation in the mammary glands of dairy cows. PLoS ONE.

[45] Hennighausen L., Robinson G.W., Wagner K.-U., Liu X. (1997). Prolactin signaling in mammary gland development. J. Biol. Chem..

[46] Robinson S.D., Roberts A.B., Daniel C.W. (1993). TGF β suppresses casein synthesis in mouse mammary explants and may play a role in controlling milk levels during pregnancy. J. Cell. Biol..

[47] Saito S., Yoshida M., Ichijo M., Ishizaka S., TSUJH T. (1993). Transforming growth factor-β (TGF-β) in human milk. Clin. Exp. Immunol..

[48] Hobert O. (2004). Common logic of transcription factor and microRNA action. Trends Biochem. Sci..

[49] Shalgi R., Lieber D., Oren M., Pilpel Y. (2007). Global and local architecture of the mammalian microRNA–transcription factor regulatory network. PLoS Comput. Biol..

[50] Hobert O. (2008). Gene regulation by transcription factors and microRNAs. Science.

[51] Barbera J.M., Clements M., Thomas P., Rodriguez T., Meloy D., Kioussis D., Beddington R. (2000). The homeobox gene Hex is required in definitive endodermal tissues for normal forebrain, liver and thyroid formation. Development.

[52] Puppin C., Puglisi F., Pellizzari L., Manfioletti G., Pestrin M., Pandolfi M., Piga A., Di Loreto C., Damante G. (2006). HEX expression and localization in normal mammary gland and breast carcinoma. BMC Cancer.

[53] Fu M., Wang C., Li Z., Sakamaki T., Pestell R.G. (2004). Minireview: Cyclin D1: Normal and abnormal functions. Endocrinology.

[54] Morey L., Aloia L., Cozzuto L., Benitah S.A., Di Croce L. (2013). RYBP and Cbx7 define specific biological functions of polycomb complexes in mouse embryonic stem cells. Cell. Rep..

[55] Livingstone L.R., White A., Sprouse J., Livanos E., Jacks T., Tlsty T.D. (1992). Altered cell cycle arrest and gene amplification potential accompany loss of wild-type p53. Cell.

[56] Humphreys K.J., McKinnon R.A., Michael M.Z. (2014). MiR-18a inhibits CDC42 and plays a tumour suppressor role in colorectal cancer cells. PLoS ONE.

[57] Mogilyansky E., Rigoutsos I. (2013). The miR-17/92 cluster: A comprehensive update on its genomics, genetics, functions and increasingly important and numerous roles in health and disease. Cell. Death Differ..

[58] Wu W., Takanashi M., Borjigin N., Ohno S., Fujita K., Hoshino S., Osaka Y., Tsuchida A., Kuroda M. (2013). MicroRNA-18a modulates STAT3 activity through negative regulation of PIAS3 during gastric adenocarcinogenesis. Br. J. Cancer.

[59] Cai Y., Chen H., Jin L., You Y., Shen J. (2013). STAT3-dependent transactivation of miRNA genes following Toxoplasma gondii infection in macrophage. Parasit. Vectors.

[60] Brock M., Trenkmann M., Gay R.E., Michel B.A., Gay S., Fischler M., Ulrich S., Speich R., Huber L.C. (2009). Interleukin-6 modulates the expression of the bone morphogenic protein receptor type II through a novel STAT3-microRNA cluster 17/92 pathway. Circ. Res..

[61] Rao X., Di Leva G., Li M., Fang F., Devlin C., Hartman-Frey C., Burow M.E., Ivan M., Croce C.M., Nephew K.P. (2011). MicroRNA-221/222 confers breast cancer fulvestrant resistance by regulating multiple signaling pathways. Oncogene.

[62] Hwang M.S., Yu N., Stinson S.Y., Yue P., Newman R.J., Allan B.B., Dornan D. (2013). MiR-221/222 targets adiponectin receptor 1 to promote the epithelial-to-mesenchymal transition in breast cancer. PLoS ONE.

[63] Ye X., Bai W., Zhu H., Zhang X., Chen Y., Wang L., Yang A., Zhao J., Jia L. (2014). MiR-221 promotes trastuzumab-resistance and metastasis in HER2-positive breast cancers by targeting PTEN. BMB Rep..

[64] Dupont J., Renou J.P., Shani M., Hennighausen L., LeRoith D. (2002). PTEN overexpression suppresses proliferation and differentiation and enhances apoptosis of the mouse mammary epithelium. J. Clin. Investig..

[65] Chen C.-C., Stairs D.B., Boxer R.B., Belka G.K., Horseman N.D., Alvarez J.V., Chodosh L.A. (2012). Autocrine prolactin induced by the Pten–Akt pathway is required for lactation initiation and provides a direct link between the Akt and STAT5 pathways. Genes Dev..

[66] Carbon S., Ireland A., Mungall C.J., Shu S., Marshall B., Lewis S., Group W.P.W. (2009). AmiGO: Online access to ontology and annotation data. Bioinformatics.

[67] Wickramasinghe S., Rincon G., Islas-Trejo A., Medrano J.F. (2012). Transcriptional profiling of bovine milk using RNA sequencing. BMC Genom..

[68] Willets J.M., Brighton P.J., Mistry R., Morris G.E., Konje J.C., Challiss R.J. (2009). Regulation of oxytocin receptor responsiveness by G protein-coupled receptor kinase 6 in human myometrial smooth muscle. Mol. Endocrinol..

[69] Neville M.C., McFadden T.B., Forsyth I. (2002). Hormonal regulation of mammary differentiation and milk secretion. J. Mammary Gland Biol. Neoplasia.

[70] Lefcourt A.M., Akers R.M. (1983). Is oxytocin really necessary for efficient milk removal in dairy cows?. J. Dairy Sci..

[71] Armstrong D., Hansel W. (1959). Alteration of the bovine estrous cycle with oxytocin. J. Dairy Sci..

[72] Politi K., Feirt N., Kitajewski J. (2004). Notch in mammary gland development and breast cancer. Semin. Cancer Biol..

[73] Yalcin-Ozuysal Ö., Fiche M., Guitierrez M., Wagner K.-U., Raffoul W., Brisken C. (2010). Antagonistic roles of Notch and p63 in controlling mammary epithelial cell fates. Cell. Death Differ..

[74] Tiwari N., Tiwari V.K., Waldmeier L., Balwierz P.J., Arnold P., Pachkov M., Meyer-Schaller N., Schübeler D., van Nimwegen E., Christofori G. (2013). Sox4 Is a master regulator of epithelial-mesenchymal transition by controlling EZH2 expression and epigenetic reprogramming. Cancer Cell..

[75] Lapébie P., Borchiellini C., Houliston E. (2011). Dissecting the PCP pathway: One or more pathways?. Bioessays.

[76] Wansleeben C., Meijlink F. (2011). The planar cell polarity pathway in vertebrate development. Dev. Dynam..

[77] Cortijo C., Gouzi M., Tissir F., Grapin-Botton A. (2012). Planar cell polarity controls pancreatic β cell differentiation and glucose homeostasis. Cell. Rep..

[78] Walck-Shannon E., Hardin J. (2014). Cell intercalation from top to bottom. Nat. Rev. Mol. Cell. Biol..

[79] Oh I.-H., Reddy E.P. (1999). The *myb* gene family in cell growth, differentiation and apoptosis. Oncogene.

[80] Lai E.C., Wiel C., Rubin G.M. (2004). Complementary miRNA pairs suggest a regulatory role for miRNA: miRNA duplexes. RNA.

[81] He L., Hannon G.J. (2004). MicroRNAs: Small RNAs with a big role in gene regulation. Nat. Rev. Genet..

[82] Carraro G., Shrestha A., Rostkovius J., Contreras A., Chao C.-M., El Agha E., MacKenzie B., Dilai S., Guidolin D., Taketo M.M. (2014). MiR-142–3p balances proliferation and differentiation of mesenchymal cells during lung development. Development.

[83] Sun W., Shen W., Yang S., Hu F., Li H., Zhu T.-H. (2010). MiR-223 and miR-142 attenuate hematopoietic cell proliferation, and miR-223 positively regulates miR-142 through LMO2 isoforms and CEBP-β. Cell. Res..

[84] Chapman R.S., Lourenco P., Tonner E., Flint D., Selbert S., Takeda K., Akira S., Clarke A.R., Watson C.J. (2002). The role of STAT3 in apoptosis and mammary gland involution. Biol. Mammary Gland.

[85] Philp J.A., Burdon T.G., Watson C.J. (1996). Differential activation of STATs 3 and 5 during mammary gland development. FEBS Lett..

[86] Anderson S.T., Barclay J.L., Fanning K.J., Kusters D.H., Waters M.J., Curlewis J.D. (2006). Mechanisms underlying the diminished sensitivity to prolactin negative feedback during lactation: Reduced STAT5 signaling and up-regulation of cytokine-inducible SH2 domain-containing protein (CIS) expression in tuberoinfundibular dopaminergic neurons. Endocrinology.

[87] Reichenstein M., Rauner G., Barash I. (2011). Conditional repression of STAT5 expression during lactation reveals its exclusive roles in mammary gland morphology, milk-protein gene expression, and neonate growth. Mol. Reprod. Dev..

[88] Gallego M.I., Binart N., Robinson G.W., Okagaki R., Coschigano K.T., Perry J., Kopchick J.J., Oka T., Kelly P.A., Hennighausen L. (2001). Prolactin, growth hormone, and epidermal growth factor activate STAT5 in different compartments of mammary tissue and exert different and overlapping developmental effects. Dev. Biol..

[89] Barash I. (2006). STAT5 in the mammary gland: Controlling normal development and cancer. J. Cell. Phys..

[90] Friedlander M.R., Chen W., Adamidi C., Maaskola J., Einspanier R., Knespel S., Rajewsky N. (2008). Discovering microRNAs from deep sequencing data using miRDeep. Nat. Biotechnol..

[91] Love M.I., Huber W., Anders S. (2014). Moderated estimation of fold change and dispersion for RNA-seq data with DESeq2. Genome Biol..

[92] Langfelder P., Horvath S. (2008). WGCNA: An R package for weighted correlation network analysis. BMC Bioinform..

[93] Langfelder P., Zhang B., Horvath S. (2008). Defining clusters from a hierarchical cluster tree: The Dynamic Tree Cut package for R. Bioinformatics.

[94] Fuller T.F., Ghazalpour A., Aten J.E., Drake T.A., Lusis A.J., Horvath S. (2007). Weighted gene coexpression network analysis strategies applied to mouse weight. Mamm. Genome.

[95] Bindea G., Mlecnik B., Hackl H., Charoentong P., Tosolini M., Kirilovsky A., Fridman W.-H., Pagès F., Trajanoski Z., Galon J. (2009). ClueGO: A Cytoscape plug-in to decipher functionally grouped gene ontology and pathway annotation networks. Bioinformatics.

