# Supplementary material for: Co-Expression Network and Pathway Analyses Reveal Important Modules of miRNAs Regulating Milk Yield and Component Traits
Source: Int J Mol Sci. 2017 Jul 18;18(7):1560. doi: 10.3390/ijms18071560 (PMC5536048; doi:10.3390/ijms18071560)
Supplement: Supplementary file 1 [file ijms-18-01560-s001.zip › ijms-208418-supplementary-layout Nicole.pdf]

Supplementary Materials: Co-Expression Network and Pathway Analyses Reveal Important Modules of miRNAs Regulating Milk Yield and Component Traits

Duy N. Do, Pier-Luc Dudemaine, Ran Li and Eveline M. Ibeagha-Awemu

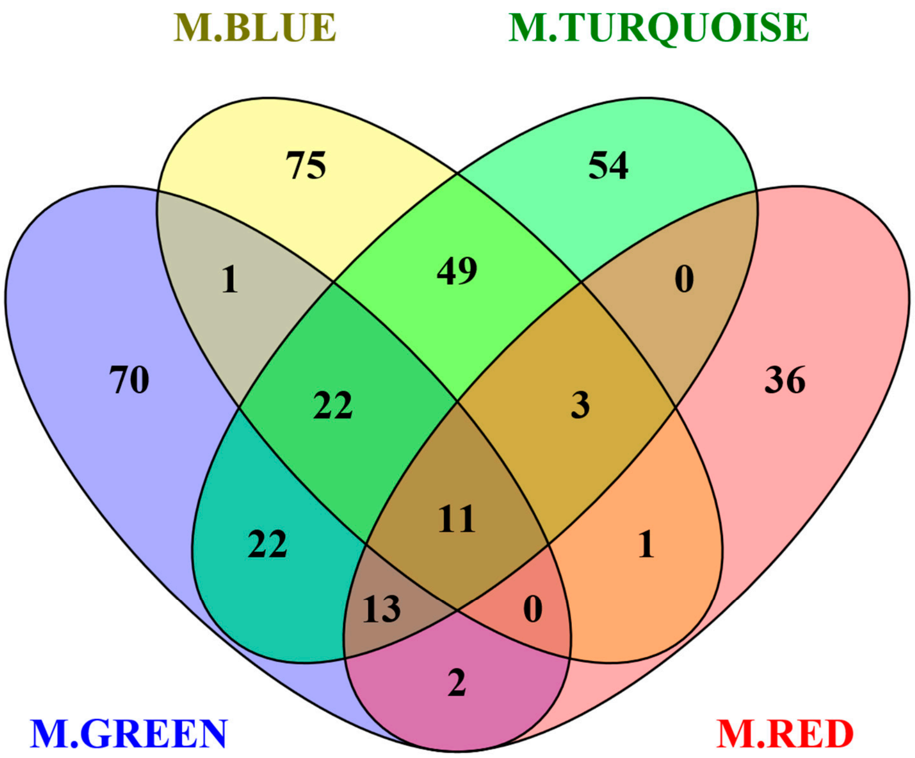

Figure S1. Venn dendrogram showing numbers of overlapped gene ontology terms significantly enriched for target genes of miRNAs in the GREEN, BLUE, TURQUOISE and RED modules.

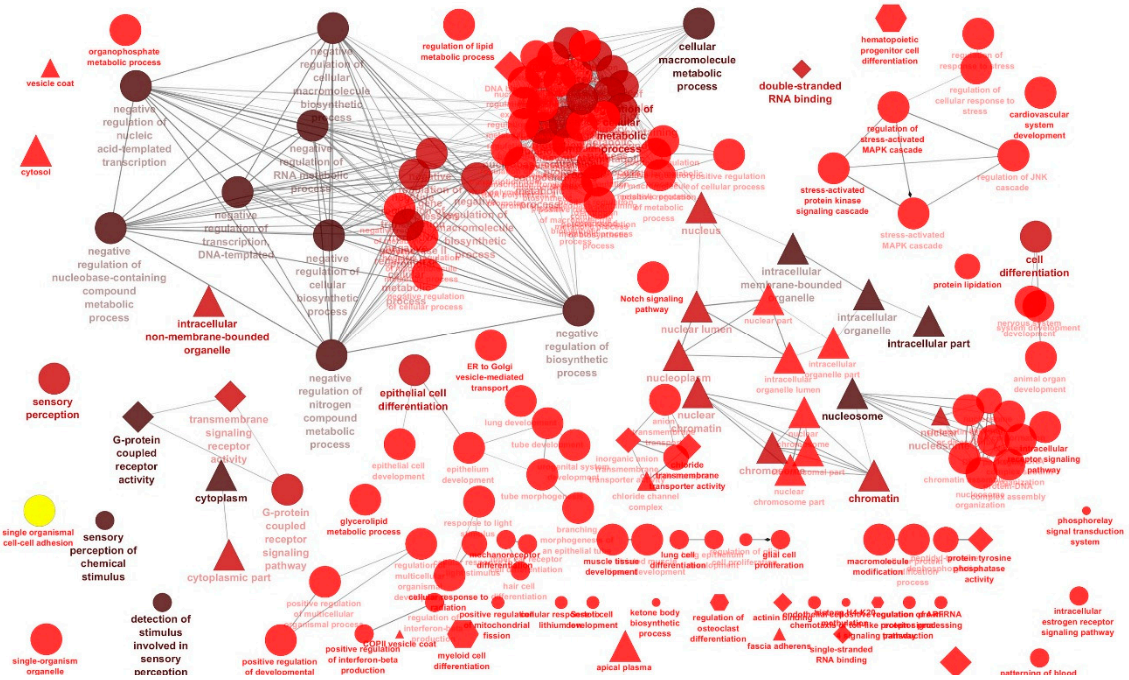

Figure S2. Enriched gene ontology terms for target genes of miRNAs in the BLUE module. The round, triangle and diamond shapes present biological process, cellular component and molecular function gene ontology terms, respectively.

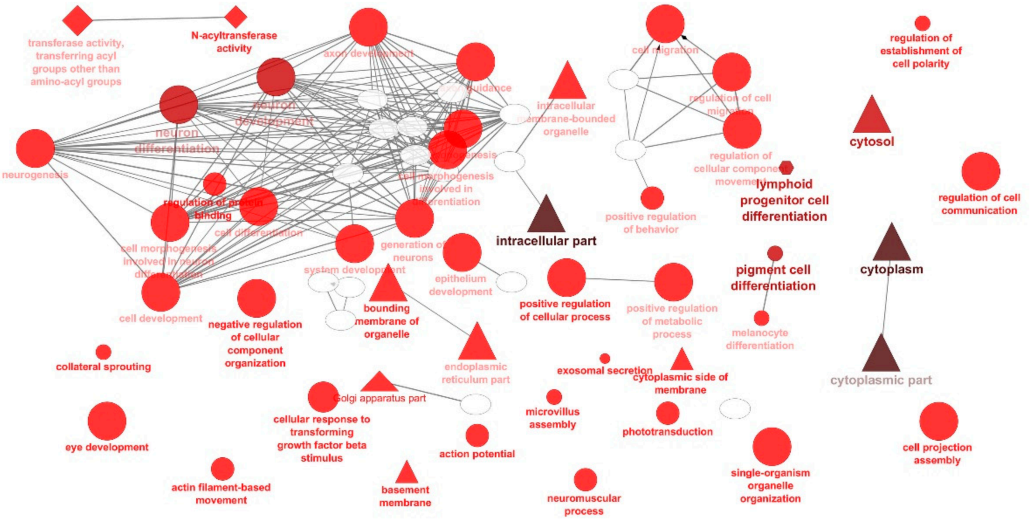

Figure S3. Enriched gene ontology terms for target genes of miRNAs in the RED module. The round, triangle and diamond shapes present biological process, cellular component and molecular function gene ontology terms, respectively.

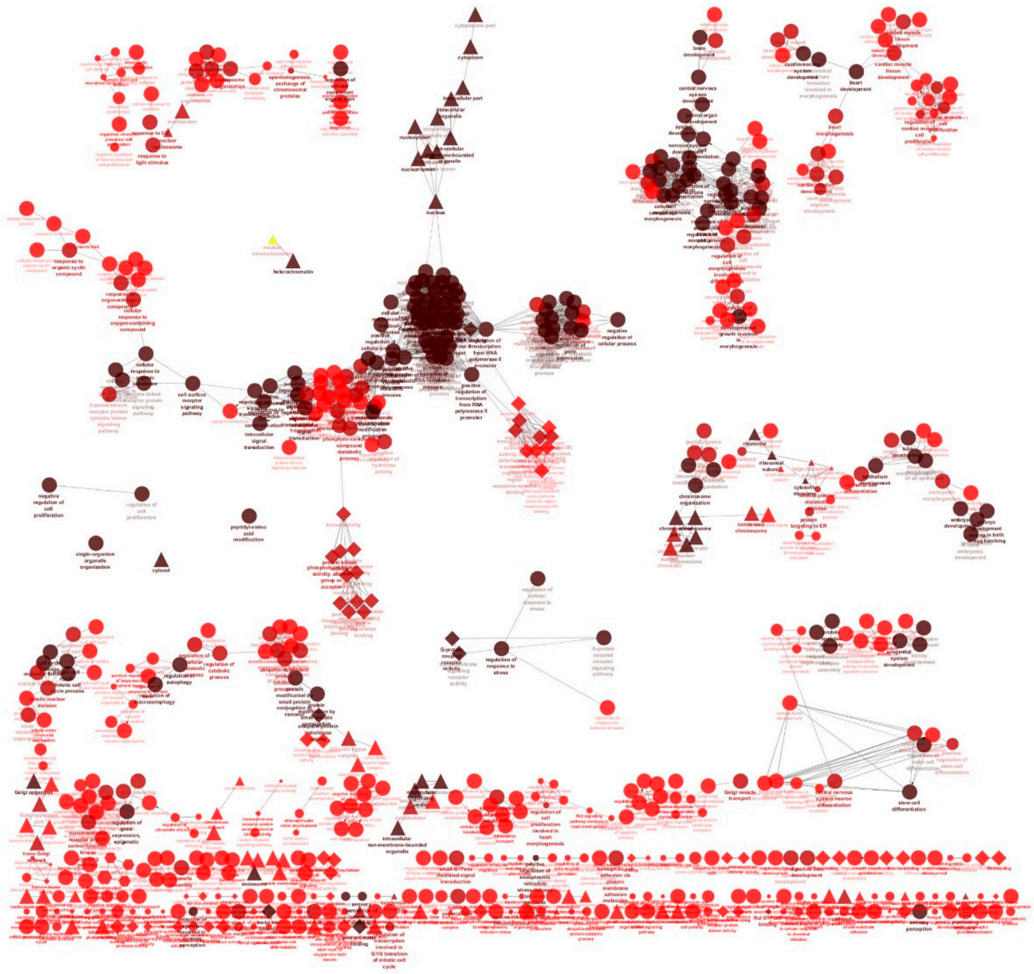

Figure S4. Enriched gene ontology terms for target genes of miRNAs in the TURQUOISE module. The round, triangle and diamond shapes present biological process, cellular component and molecular function gene ontology terms, respectively.

Table S1. Predicted target genes for miRNA members of the (a) GREEN, (b) BLUE, (c) RED and (d) TURQUOISE modules; **Table S2.** Gene ontologies enriched for target genes of miRNAs in the (a) GREEN, (b) RED, (c) BLUE and (d) TURQUOISE modules; **Table S3.** Signaling pathways enriched for target genes of miRNAs in *the* (a) GREEN, (b) BLUE, (c) TURQUOISE and (d) RED modules; Table S4: Upstream transcription regulators enriched for target genes of miRNAs in each module.

Please fine Table S1–S3 in the supplementary material.
